# 

**Published:** 1873-08

**Authors:** 


### TO READERS AND CORRESPONDENTS.

Communications intended for publication, and exchange journals and pape[illegible] should be addressed to the EDITOR OF THE AMERICAN JOURNAL OF DENT[illegible] SCIENCE, 259 N. Eutaw St., Baltimore, Md. All letters relating to the busine[illegible] of the journal, or containing cash remittances, to be directed to Messrs. SNOWDE[illegible] & COWMAN. Articles for publication should be received by the editor at lea[illegible] three weeks previous to the first day of the month on which the number in whic[illegible] it is designed for them to appear, is issued.

### CONTENTS:

### ORIGINAL COMMUNICATIONS.

☞ The American Journal of Dental Science will contain fifty pages o[illegible] reading matter in each number, making a volume of not less than

*Six Hundred* pages

**EXCLUSIVE OF ADVERTISEMENTS.**

### THE AMERICAN JOURNAL OF DENTAL SCIENCE.

F. J. S. GORGAS, M. D., D. D. S., Editor.

The *Journal* is issued on the first of each month and contains not less than *fifty* pages of reading matter in each number, exclusive of advertisements, making a volume of *six hundred* pages per annum.

In each number will be found Original Communications, Correspondence, Selected Articles, Monthly Summary, Bibliographical Notices and Editorial Matter, and every effort will be exerted to render the *Journal* worthy of the patronage of the Dental profession.

A generous co-operation is respectfully solicited from the members of the profession; and as the *Journal* has now a printing office of its own which materially lessens the cost of its publication, it will be issued at the reduced subscription price of

**$2.50 Per Annum in Advance.**

Communications are respectfully solicited on all subjects pertaining to the practice of dentistry, as well as reports of cases occurring in dental practice.

### RATES OF ADVERTISING.

| | 1 MO. | 2 MO. | 3 MO. | 6 MO. | 12 MO. |
|---|---|---|---|---|---|
| 1 PAGE. | $15 00 | $22 50 | $30 00 | $50 00 | $100 00 |
| ½ " | 10 00 | 15 00 | 20 00 | 30 00 | 50 00 |
| ¼ " | 7 00 | 11 00 | 15 00 | 20 00 | 30 00 |
| ⅛ " | 5 00 | 8 00 | 11 00 | 15 00 | 20 00 |

All original communications designed for publication, works for review, new instruments for notice, exchanges, &c., should be directed to the editor, 259 N. Eutaw St., Baltimore, Md.

All advertisements and subscriptions should be addressed to

SNOWDEN & COWMAN,

*Publishers of the Am. Journal of Dental Science.*

86 W. FAYETTE ST. BET. CHARLES & LIBERTY STS.,

BALTIMORE, Md.

